# Blood pressure lowering treatment for preventing stroke recurrence: a systematic review and meta-analysis

**DOI:** 10.1186/1755-7682-2-30

**Published:** 2009-10-20

**Authors:** Shaheen E Lakhan, Michael T Sapko

**Affiliations:** 1 Global Neuroscience Initiative Foundation, Los Angeles, California, USA

## Abstract

**Background:**

While hypertension is a leading risk factor for an initial stroke, the role of blood pressure lowering to prevent subsequent stroke is less clear. The results of recent large clinical trials investigating effects of antihypertensive agents in patients with a history of stroke have not shown a significant benefit; findings that are at odds with previous data. Our meta-analysis systematically evaluates the available, relevant trials to examine the role of antihypertensive drugs in preventing recurrent stroke.

**Methods:**

MEDLINE, CENTRAL, and ClinicalTrials.gov were systematically searched and bibliographies from key reports were examined. All randomized, placebo-controlled trials that tested blood pressure lowering agents in patients with stroke or transient ischemic attack were identified. The results from these trials were combined and meta-analyses were performed.

**Results:**

Ten studies were found to contain relevant endpoints and presented data allowing meta-analysis. Agents that lowered blood pressure reduced recurrent stroke (OR 0.71, 95% CI 0.59-0.86, P = 0.0004) and cardiovascular events (OR 0.69, 95% CI 0.57-0.85, P = 0.0004) in patients with a previous stroke or TIA. These agents did not affect the rate of myocardial infarction (OR 0.86, 95% CI 0.73-1.01, P = 0.07) or all-cause mortality (OR 0.95, 95% CI 0.83-1.07, P = 0.39) in this patient population.

**Conclusion:**

Despite recent large trials showing no significant effect, in patients that have experienced a TIA or stroke, blood pressure lowering agents reduced the occurrence of subsequent stroke and cardiovascular events. The rate of myocardial infarction and all-cause mortality was unchanged.

## Background

Hypertension is the leading risk factor for cerebrovascular accident [1] and increases the risk of stroke sevenfold [2]. While primary prevention of stroke must include adequate blood pressure (BP) control, BP control in the secondary prevention of stroke is less clear. Individuals who suffer a stroke or transient ischemic attack (TIA) are at increased risk for another cardiovascular event [3]. While lowering BP during an acute cerebrovascular accident remains controversial, there may be a benefit to lowering blood pressure after the acute phase of the event has passed. Meta-analyses indicate that BP reduction can decrease the risk of recurrent stroke by one third [4]. In primary stroke prevention, BP is strongly associated with stroke risk and association is continuous down to pressures of 115/75 mmHg [5]. In secondary stroke prevention, there has been concern that too great a BP reduction can interfere with cerebral blood flow and can lead to ischemia. In fact, earlier cohort studies seemed to show a J-shaped curve of blood pressure and stroke recurrence [6,7]. However, recent studies such as HOPE [8,9] and PROGRESS [10,11] have failed to demonstrate this negative association at lower BP.

Rashid and coauthors performed a systematic review of blood pressure and recurrent stroke in 2003 [12] and concluded that for patients with previous stroke or TIA, blood-pressure lowering agents reduced the risk of subsequent cardiovascular events. Furthermore, the authors report that blood pressure reduction is negatively associated with recurrent stroke and myocardial infarction. Since 2003 however, results from large randomized controlled trials have appeared to contradict this finding, specifically the recent study using the angiotensin receptor blocker telmisartan [13] and the interpretation of the PROGRESS trial [14]. Our current investigation provides an updated systematic review of the literature with meta-analysis examining the role of BP reduction and the use of antihypertensive agents to prevent the recurrence of stroke.

## Methods

### Identification, Inclusion and Exclusion

**Figure 1:**
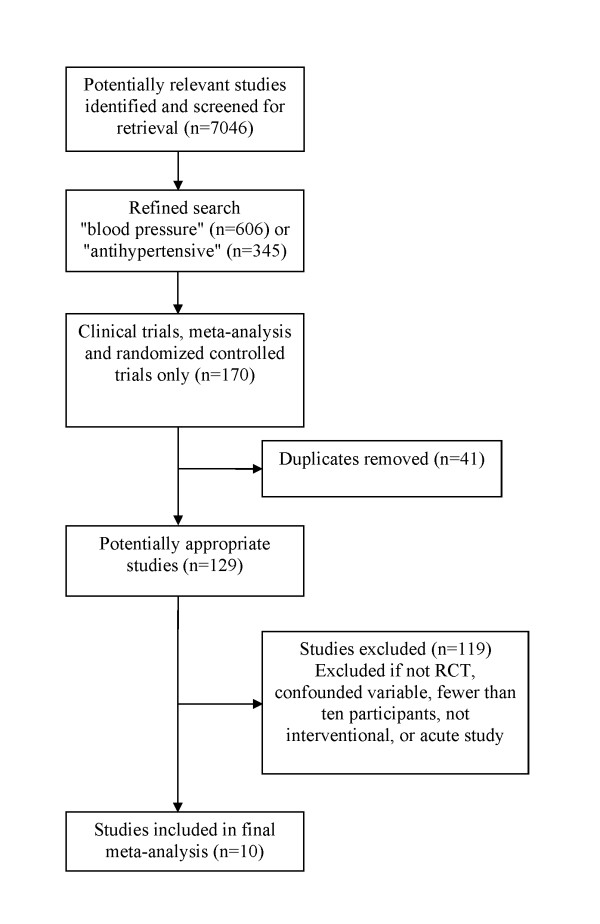
**Flow diagram of included studies**.

Clinical trials were identified using MEDLINE/PubMed and CENTRAL (Cochrane Central Register of Controlled Trials) in April 2009. Literature searches were performed using the following search terms: "recurrence" OR "recurrent" OR "secondary prevention" AND "stroke." These search terms resulted in 7,046 primary articles from the PubMed database, including 1,702 review articles (see Figure 1 for a flow diagram). To these terms were added either "blood pressure" or "antihypertensive" which reduced the number of entries to 606 and 345 articles respectively. When these results were limited to clinical trials, meta-analysis and randomized controlled trials, the number of articles decreased to 119 for "blood pressure" and 51 for "antihypertensive." The resulting articles were then manually screened and duplicates removed. To account for the possibility that the PubMed search excluded one or more useful studies, the authors surveyed the bibliographies of relevant articles and reviews for additional articles that were not returned in the database search. Also, the same search terms were used in the ClinicalTrials.gov search engine to identify registered clinical trials that are complete, ongoing and currently enrolling participants.

Articles were excluded if at least one treatment group or study arm was not comprised entirely of patients with a previous stroke and/or TIA. Similarly, clinical trials were excluded if they were not compared against a randomized placebo group. In certain cases, BP was presented as part of an integrated approach to secondary stroke prevention and these articles were excluded from our analysis. Clinical trials that studied the effects of blood pressure lowering agents during acute stroke were also excluded. [See Additional file 1 for a quality of reporting of meta-analyses (QUOROM) statement checklist.]

### Data Abstraction

Data were extracted independently by the authors and any disagreements were resolved by consensus.

### Clinical Outcomes

The clinical trials surveyed in this meta-analysis reported various primary, secondary and tertiary endpoints. The endpoints of interest for our analysis were stroke recurrence, cardiovascular event, myocardial infarction and all-cause mortality. The stroke recurrence endpoint included both fatal and non-fatal stroke. For the purposes of this analysis, the term "stroke" included both ischemic stroke and intracerebral hemorrhage, although these subgroups are identified in the text when possible. The myocardial infarction endpoint included fatal and non-fatal events unless otherwise noted. Cardiovascular event, the endpoint used in the HOPE [8] and PRoFESS trials [13], includes death from a cardiovascular cause, recurrent stroke or myocardial infarction.

### Statistical Analysis

Individual data including the number of participants experiencing a clinical outcome and the total number of study participants for each arm were extracted to determine odds ratios for each study or subgroup. These data were analyzed using Review Manager (RevMan) version 5.0 [15]. The Mantel-Haenszel method was used for all odds ratio analyses and all data were dichotomous. We assumed that significant statistical heterogeneity existed among the trials studies given their low number and the large differences between studies. A random effects model was used for all analyses. Inconsistency was determined by chi-square testing and determination of the I^2 ^statistic [16]. Putative heterogeneity was identified by I^2 ^values above 40% and a significant Chi square statistic of P < 0.1. Where significant heterogeneity existed, a random effects model was used and we attempted to identify sources of heterogeneity. Funnel plots were constructed [17] to identify publication bias among studies. Ninety-five percent confidence intervals were used throughout and P values less than 0.05 were considered statistically significant.

## Results

Ten randomized clinical trials were identified as having study participants with prior TIA, stroke or "reversible ischemic neurologic defect" who were chronically treated with a blood pressure lowering agent [8,10,13,18-24]. The summary of included trials is shown in Table 1. Some analyses did not include all ten trials because the clinical endpoint could not be clearly elucidated from the text [25-32]. Excluded trials are listed in Table 2.

**Table 1:** Characteristics of randomized clinical trials investigating the role of blood pressure lowering on stroke recurrence.

**Study**	**Year**	**Drug**	**Type of Stroke**	**Subjects (Treated, Control)**	**Mean Follow-up Period (years)**	**Decrease in BP with Treatment (sys/dia in mmHg)**
**BPLPRS **[23]	2005	Perindopril + indapamide vs. placebo	TIA, Isch, Hem	1520 (762, 758)	4	14/6

**Dutch TIA **[20]	1993	Atenolol vs. placebo	TIA or non-disabling Isch	1473 (732, 741)	2.6	5.8/2.9

**HOPE **[33]	2000	Ramipril vs. placebo	TIA or stroke NS	1013 (500, 513)	5	NS

**HSCSG **[18]	1974	Deserpidine + methyclothiazide vs. placebo	TIA, Isch, Hem	452 (233, 219)	2.8	25/12.3

**Martí Massó **[19]	1990	Nicardipine vs. nothing	TIA, RIND, Isch	264 (170, 94)	1	NS

**PATS **[21]	1995	Indapamide vs. placebo	TIA, Isch, Hem	5665 (2841, 2824)	2.8	6.2/2.9

**PRoFESS **[13]	2008	Telmisartan vs. placebo	Isch	20332 (10146, 10186)	2.5	3.8/2

**PROGRESS **[10]	2001	Perindopril + indapamide OR perindopril vs. placebo	TIA, Isch, Hem	6105 (3051, 3054)	4	9/4

		Perindopril vs. placebo		2561^a ^(1281, 1280)		5/3

		Perindopril + indapamide vs. placebo		3544^a ^(1770, 1774)		12/5

**SCOPE **[24]	2005	Candesartan vs. placebo	NS	193 (96, 97)	3.7	-0.4/1.5^b^

**TEST **[22]	1995	Atenolol vs. placebo	NS	720 (372, 348)	2.5	4/3

Hem, hemorrhagic stroke or intracerebral hemorrhage; Isch, ischemic stroke; NS, not specified in published report; RIND, reversible ischemic neurological defect; TIA, transient ischemic attack.

**a **Part of the original 6,105 PROGRESS study participants.

**b **Mean systolic blood pressure increased in treatment group.

**Table 2:** Selected trials identified by literature search and reasons for exclusion.

**Study**	**Reason for Exclusion**
**ACCESS **[27]	Acute study, no control

**Kaplan **[31]	Observational, not randomized or interventional

**Lehigh Valley **[26]	Not randomized or interventional

**MOSES **[30]	Not placebo controlled, compared two antihypertensives

**Ribas Mundó **[25]	Not randomized or placebo controlled

**PEACE **[28]	Less than ten stroke survivors per arm

**SAPSI **[29]	Not randomized or interventional

**VALUE **[32]	Not placebo controlled, compared two antihypertensives

**Figure 2:**
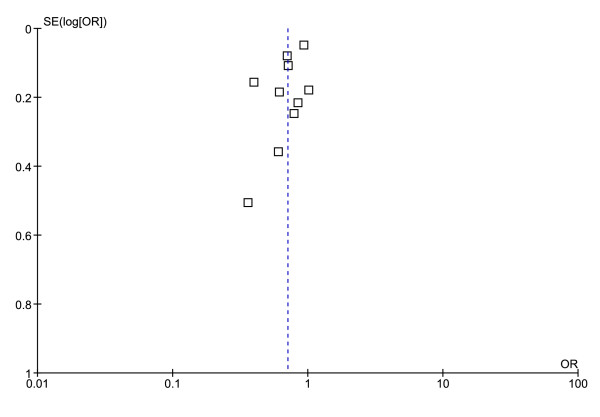
**Funnel plot comparison of studies examining the effect of blood pressure lowering agents on the risk of recurrent stroke**. One study, BPLPRS, fell outside of the 95% confidence region.

All ten studies reported stroke recurrence as a primary or secondary endpoint. The total number of patients included in this analysis was 37,737 of which 18,903 received active treatment, 18,740 received placebo, and 94 received nothing. The Martí Massó study [19] was the only open trial included in the analysis. Lowering blood pressure significantly decreased the odds ratio of stroke recurrence shown in Additional file 2 (OR 0.71, 95% CI 0.59-0.86, P = 0.0004). Tests [16] showed that the ten studies analyzed had a considerable degree of heterogeneity (P < 0.01, I^2 ^= 78%). Along the ten studies there were variations in study population. These variations included age, race, time of follow-up, antihypertensive medication used and degree of hypertension at trial start. Only one study, BPLPRS [23], lead to significant funnel plot asymmetry (Figure 2). When this study was eliminated from the analysis, the blood pressure lowering was still associated with a reduced risk of recurrent stroke though the effect was less pronounced (OR 0.77, 95% CI 0.66-0.90, P = 0.0009). BPLPRS studied the effect of ACE inhibitor, perindopril, combined with a diuretic, indapamide, on recurrent stroke in Chinese patients exclusively.

Cardiovascular event is a composite endpoint which included death from any vascular cause, non-fatal stroke and non-fatal myocardial infarction. This composite was used in the HOPE trial [33] and has been adopted by authors in subsequent trials [13]. Random effects analysis [see Additional file 3] showed that regardless of the agent used, blood pressure reduction reduced the number of cardiovascular events (OR 0.69, 95% CI 0.57-0.85, P = 0.0004). The Martí Massó study [19] was not used in the meta-analysis because the cardiovascular event endpoint could not be determined from the published report. The number of subjects was 18,733 for treated groups and 18,740 for placebo. Heterogeneity was again quite high and likely due to different antihypertensive medications used among the studies.

The relative risks of myocardial infarction and all-cause mortality were determined from eight and seven of the ten trials, respectively. The SCOPE [24] and Martí Massó [19] studies were omitted from the myocardial infarction analysis and the HOPE [8], PATS [21] and SCOPE [24] studies were omitted from the all-cause mortality analysis because data regarding the respective endpoint could not be obtained. Despite these omissions each analysis included over 15,000 patients. There were 18,637 patients in the treatment arm and 18,638 patients in the placebo arm for the myocardial infarction endpoint and 15,466 in the treated arm and 15,400 in the placebo arm for all-cause mortality. Tests of heterogeneity and inconsistency were relatively low for both analyses (Myocardial infarction, P = 0.24, I^2 ^= 24%; All-cause mortality, P = 0.21, I^2 ^= 29%). As shown in Additional file 4, blood pressure lowering agents did not show a statistically significant effect on myocardial infarction in patients that had experienced a stroke or TIA (OR 0.86, 95% CI 0.73-1.01, P = 0.07). The all-cause mortality rate was similar between both active and placebo treated patients (OR 0.95, 95% CI 0.83-1.07, P = 0.39). Additional file 5 shows the forest plot of studies that included explicit all-cause mortality data.

## Discussion

Results from recent large randomized clinical trials [13] have called into question the role of blood pressure lowering agents in patients with previous stroke. Stroke survivors usually have significant pill burdens [34] making judicious medication selection an important part of the health care plan. The number of randomized, prospective studies that specifically examine the role of blood pressure control in patients that have already experienced a stroke or TIA is relatively low [35]. The results of systematic review and meta-analysis show that blood pressure lowering medications confer significant benefit to stroke and TIA survivors by reducing the rate of subsequent strokes and major cardiovascular events. This is especially important given that between 8 and 15 percent of stroke survivors will experience a second event within the first year [36] and the recurrent events lead more often to disability and death [37].

The findings of our analysis are in line with a previous meta-analysis of the subject done by Rashid and Bath in 2003 [12]. They found that all cases of recurrent stroke and non-fatal stroke were fewer in the antihypertensive treated group. In contrast to the Rashid meta-analysis, blood pressure lowering agents in patients that had had a previous stroke did not alter the rate of subsequent myocardial infarction in our analysis. This finding is likely due to the results of the PRoFESS trial [13] which were not available in 2003. The PRoFESS trial reports essentially identical rates of myocardial infarction in both the treatment and placebo groups. Because of the size of the trial, it was given the largest share of relative weight (28.3%) of the studies we analyzed [see Additional file 4]. When this trial was omitted from our current analysis, pharmacological blood pressure treatment conferred a statistically significant benefit for myocardial infarction; however there is no reason to omit the participants of this large, high-quality study. Thus in patients that have experienced a stroke or TIA, blood pressure reduction is not a major determinant of myocardial infarction risk. However, the overall cardiovascular health of stroke patients should not be neglected.

As shown in our analysis, blood pressure reduction in this patient population does not reduce all-cause mortality. It does, however, reduce cardiovascular events, and mortality from vascular causes is part of the composite endpoint. Information regarding cardiovascular mortality was only available for four out of the ten relevant trials and did not yield useful analysis.

The TRANSCEND trial [38] is notably missing from this meta-analysis. While not the primary focus, the TRANSCEND trial included a subset of 1,302 patients with previous ischemic stroke of which 648 and 654 were randomized to telmisartan and placebo groups, respectively. The number of relevant endpoints could not be obtained as the data is still being compiled for a forthcoming report. Nevertheless, results from the TRANSCEND study are expected to be similar to the larger PRoFESS trial in which telmisartan was also the antihypertensive.

There are always limitations to using data across trials, as opposed to data regarding individual patients. Indeed our meta-analysis does indicate inconsistencies in the data that extend beyond statistical heterogeneity, especially in the recurrent stroke and cardiovascular event groups. Some of this heterogeneity may be resolved if individual agents or drug classes were considered individually. Unfortunately due to the limited number of studies, generalizations about specific drug classes could be misleading. Studies that were excluded from our analysis because they did not include a control group could shed some light on this issue. For example, the MOSES trial [30] compared the angiotensin II receptor blocker (ARB) against the calcium channel blocker nitrendipine in patients with a history of a cerebral event. The ARB conferred enhanced benefit for the combined endpoint of cardiovascular events [30]. An important caveat regarding the MOSES trial is that a large portion of trial participants in both trial arms were on various blood pressure agents concomitantly.

Blood pressure control remains an important consideration for patients who have experienced previous TIA or stroke. Given the available data, blood pressure control does confer a benefit to stroke survivors by reducing risk of recurrent stroke and cardiovascular events. Optimal subacute and chronic treatment following stroke should be individualized to patient's needs but should include at least one blood pressure lowering agent.

## Abbreviations

ARB: angiotensin II receptor blocker; BP: blood pressure; QUOROM: quality of reporting of meta-analyses; TIA: transient ischemic attack.

## Competing interests

The authors declare that they have no competing interests.

## Authors' contributions

All authors participated in the preparation of the manuscript, and read and approved the final manuscript.

## Supplementary Material

Additional file 1 **QUOROM statement checklist**. A word document showing the QUOROM statement checklist. Click here for file

Additional file 2 **Forest plot using a random effects model showing the effect of blood pressure lowering agents on the risk of recurrent stroke**. A forest plot using a random effects model showing the effect of blood pressure lowering agents on the risk of recurrent stroke. Click here for file

Additional file 3 **Forest plot using a random effects model showing the effect of blood pressure lowering agents on the risk of a cardiovascular event**. A forest plot using a random effects model showing the effect of blood pressure lowering agents on the risk of a cardiovascular event. Click here for file

Additional file 4 **Forest plot using a fixed effects model showing the effect of blood pressure lowering agents on the risk of a myocardial infarction**. A forest plot using a fixed effects model showing the effect of blood pressure lowering agents on the risk of a myocardial infarction. Click here for file

Additional file 5 **Forest plot using a fixed effects model showing the effect of blood pressure lowering agents on the risk of all-cause mortality**. A forest plot using a fixed effects model showing the effect of blood pressure lowering agents on the risk of all-cause mortality. Click here for file
