# Supplementary material for: Blood pressure lowering treatment for preventing stroke recurrence: a systematic review and meta-analysis
Source: Int Arch Med. 2009 Oct 20;2:30. doi: 10.1186/1755-7682-2-30 (PMC2771000; doi:10.1186/1755-7682-2-30)
Supplement: Additional file 1 — QUOROM statement checklist. A word document showing the QUOROM statement checklist. [file 1755-7682-2-30-S1.DOC]

| **QUOROM Statement checklist**  **Blood pressure lowering treatment for preventing stroke recurrence: a systematic review and meta-analysis** | | | | |
| --- | --- | --- | --- | --- |
| **Heading** | **Subheading** | **Descriptor** | **Reported? (Y/N)** | **Heading: Subheading** |
| **Title** |  | Identify the report as a systematic review | Y | Title |
| **Abstract** |  | Use a structured format | Y | Abstract |
|  | Objectives | The clinical question explicitly | Y | Abstract: Background |
|  | Data sources | The databases (ie, list) and other information sources | Y | Abstract: Methods |
|  | Review methods | The selection criteria (ie, population, intervention, outcome, and study design); methods for validity assessment, data abstraction, and study characteristics, and quantitative data synthesis in sufficient detail to permit replication | Y | Abstract: Methods |
|  | Results | Characteristics of the RCTs included and excluded; qualitative and quantitative findings (ie, point estimates and confidence intervals); and subgroup analyses | Y | Abstract: Results |
|  | Conclusion | The main results | Y | Abstract: Results, Conclusion |
|  |  |  |  |  |
|  |  | **Describe** |  |  |
| **Introduction** |  | The explicit clinical problem, biological rationale for the intervention, and rationale for review | Y | Background |
| **Methods** | Searching | The information sources, in detail (eg, databases, registers, personal files, expert informants, agencies, hand-searching), and any restrictions (years considered, publication status, language of publication) | Y | Methods: Identification, Inclusion and Exclusion |
|  | Selection | The inclusion and exclusion criteria (defining population, intervention, principal outcomes, and study design | Y | Methods: Identification, Inclusion and Exclusion |
|  | Validity assessment | The criteria and process used (eg, masked conditions, quality assessment, and their findings) | Y | Methods: Identification, Inclusion and Exclusion |
|  | Data abstraction | The process or processes used (eg, completed independently, in duplicate) | Y | Methods: Data Abstraction |
|  | Study characteristics | The type of study design, participants' characteristics, details of intervention, outcome definitions, and how clinical heterogeneity was assessed | Y | Methods: Clinical Outcomes |
|  | Quantitative data synthesis | The principal measures of effect (eg, relative risk), method of combining results (statistical testing and confidence intervals), handling of missing data; how statistical heterogeneity was assessed; a rationale for any a-priori sensitivity and subgroup analyses; and any assessment of publication bias | Y | Methods: Statistical Analysis |
| **Results** | Trial flow | Provide a meta-analysis profile summarising trial flow (see figure) | Y | Methods: Identification, Inclusion and Exclusion, Figure 1 |
|  | Study characteristics | Present descriptive data for each trial (eg, age, sample size, intervention, dose, duration, follow-up period) | Y | Results: Tables 1-2 |
|  | Quantative data synthesis | Report agreement on the selection and validity assessment; present simple summary results (for each treatment group in each trial, for each primary outcome); present data needed to calculate effect sizes and confidence intervals in intention-to-treat analyses (eg 2X2 tables of counts, means and SDs, proportions) | Y | Results: Figures 2-6 |
| **Discussion** |  | Summarise key findings; discuss clinical inferences based on internal and external validity; interpret the results in light of the totality of available evidence; describe potential biases in the review process (eg, publication bias); and suggest a future research agenda | Y | Discussion |
