# Supplementary figures and images for: Blood pressure lowering treatment for preventing stroke recurrence: a systematic review and meta-analysis
Source: Int Arch Med. 2009 Oct 20;2:30. doi: 10.1186/1755-7682-2-30 (PMC2771000; doi:10.1186/1755-7682-2-30)

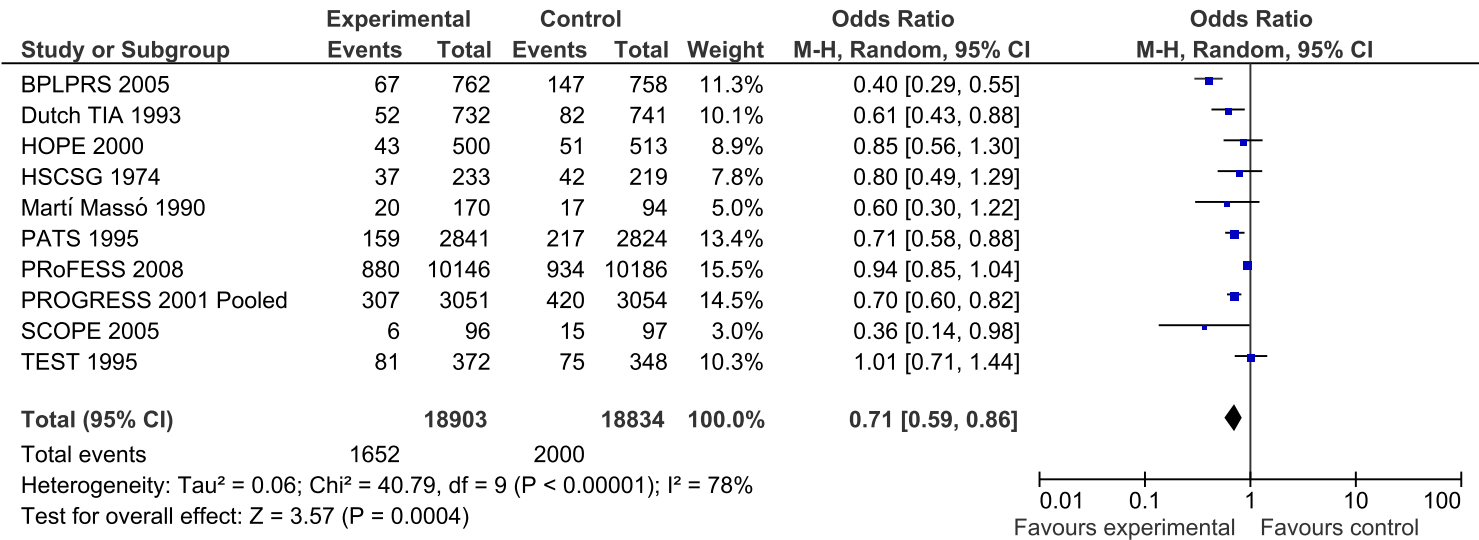

Supplement: Additional file 2 — Forest plot using a random effects model showing the effect of blood pressure lowering agents on the risk of recurrent stroke. A forest plot using a random effects model showing the effect of blood pressure lowering agents on the risk of recurrent stroke. [file 1755-7682-2-30-S2.PDF]

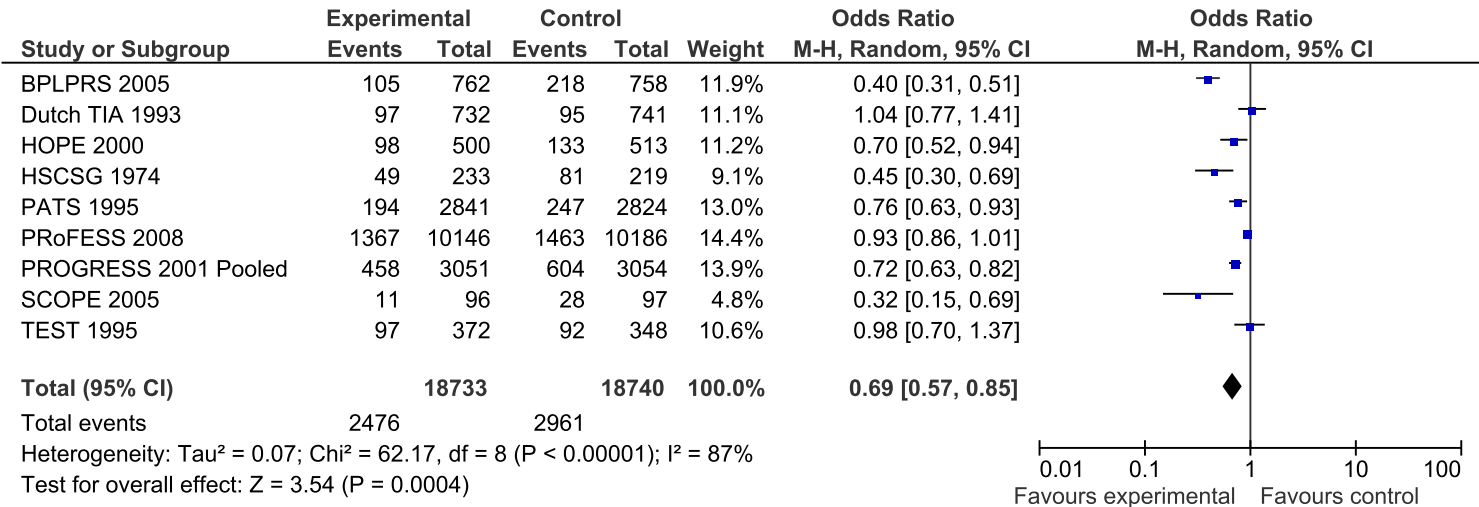

Supplement: Additional file 3 — Forest plot using a random effects model showing the effect of blood pressure lowering agents on the risk of a cardiovascular event. A forest plot using a random effects model showing the effect of blood pressure lowering agents on the risk of a cardiovascular event. [file 1755-7682-2-30-S3.PDF]

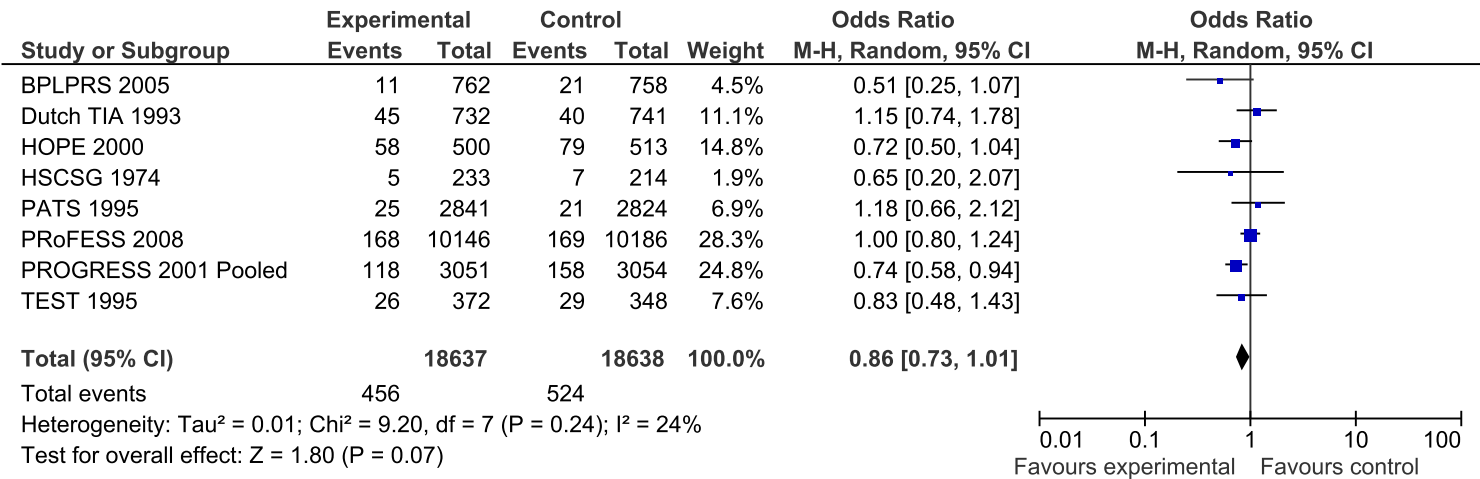

Supplement: Additional file 4 — Forest plot using a fixed effects model showing the effect of blood pressure lowering agents on the risk of a myocardial infarction. A forest plot using a fixed effects model showing the effect of blood pressure lowering agents on the risk of a myocardial infarction. [file 1755-7682-2-30-S4.PDF]

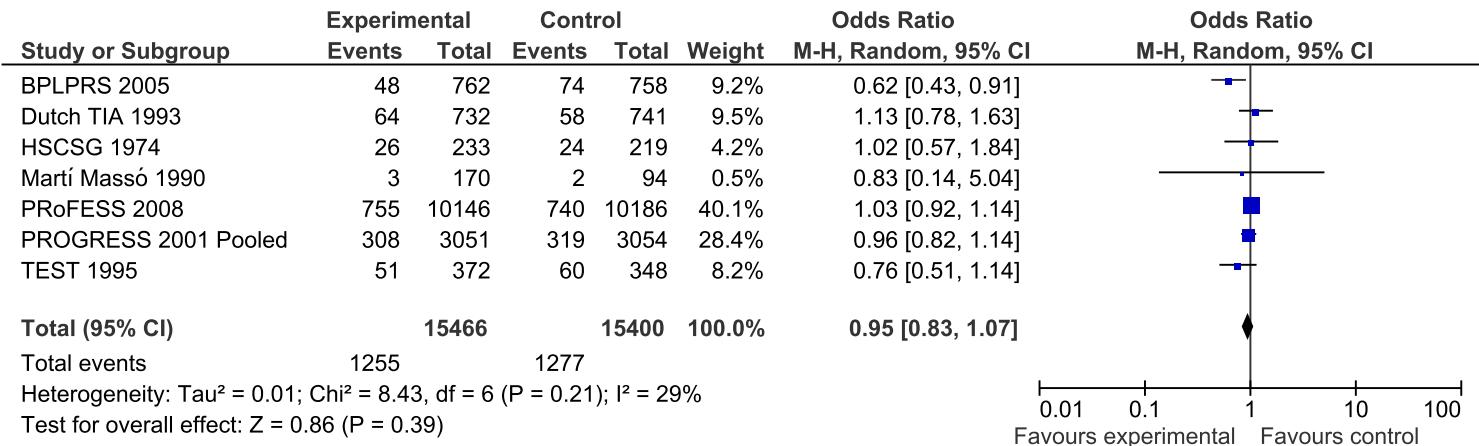

Supplement: Additional file 5 — Forest plot using a fixed effects model showing the effect of blood pressure lowering agents on the risk of all-cause mortality. A forest plot using a fixed effects model showing the effect of blood pressure lowering agents on the risk of all-cause mortality. [file 1755-7682-2-30-S5.PDF]
